# Supplementary material for: Neurodevelopmental Outcomes Associated with Early-Life Exposure to Heavy Metals: A Systematic Review
Source: Int J Environ Res Public Health. 2025 Aug 21;22(8):1308. doi: 10.3390/ijerph22081308 (PMC12386800; doi:10.3390/ijerph22081308)
Supplement: Supplementary file 1 [file ijerph-22-01308-s001.zip › Supplementary material - Questions JBI - Table S3 and Table S4.pdf]

Neurodevelopmental Outcomes Associated with Early-Life Exposure to Heavy Metals: A Systematic Review

Supplementary material 4- Questions JBI - Table S3 and Table S4

Questions: Analysis of cross-sectional studies

Q1- Were the criteria for inclusion in the sample clearly defined?
Q2- Were the study subjects and the setting described in detail?
Q3- Was the exposure measured in a valid and reliable way?
Q4- Were objective, standard criteria used for measurement of the condition?
Q5- Were confounding factors identified?
Q6- Were strategies to deal with confounding factors stated?
Q7- Were the outcomes measured in a valid and reliable way?
Q8- Was appropriate statistical analysis used?

Questions: Analysis of longitudinal studies

Q1- Were the two groups similar and recruited from the same population?
Q2- Were the exposures measured similarly to assign people to both exposed and unexposed groups?
Q3- Was the exposure measured in a valid and reliable way?
Q4- Were confounding factors identified?
Q5- Were strategies to deal with confounding factors stated?
Q6- Were the groups/participants free of the outcome at the start of the study (or at the moment of exposure)?
Q7- Were the outcomes measured in a valid and reliable way?
Q8- Was the follow up time reported and sufficient to be long enough for outcomes to occur?
Q9- Was follow up complete, and if not, were the reasons to loss to follow up described and explored?
Q10- Were strategies to address incomplete follow up utilized?
Q11- Was appropriate statistical analysis used?
