# Supplementary material for: Neurodevelopmental Outcomes Associated with Early-Life Exposure to Heavy Metals: A Systematic Review
Source: Int J Environ Res Public Health. 2025 Aug 21;22(8):1308. doi: 10.3390/ijerph22081308 (PMC12386800; doi:10.3390/ijerph22081308)
Supplement: Supplementary file 1 [file ijerph-22-01308-s001.zip › Supplementary material - Table S1.pdf]

Neurodevelopmental Outcomes Associated with Early-Life Exposure to Heavy Metals: A Systematic Review

Supplementary material - Table S1

Table S1 - Search strategies

**Database**	**Syntax**
Embase	('children'/exp OR children) AND ('heavy metals'/exp OR 'heavy metals') AND ('neurodevelopment'/exp OR neurodevelopment)
Lilacs	(children) AND (heavy metals) AND (neurodevelopment)
PsycInfo	Any Field: children *AND* Any Field: heavy metals *AND* Any Field: neurodevelopment
Pubmed	("child"[MeSH Terms] OR "child"[All Fields] OR "children"[All Fields] OR "child s"[All Fields] OR "children s"[All Fields] OR "childrens"[All Fields] OR "childs"[All Fields]) AND 'heavy metals'[All Fields] AND "neurodevelopment"[All Fields]
Scopus	(TITLE-ABS-KEY (children) AND TITLE-ABS-KEY ("heavy metals") AND TITLE-ABS-KEY (neurodevelopment))
Web of Science	children (All Fields) AND "heavy metals" (All Fields) AND neurodevelopment (All Fields)
