# Supplementary material for: Neurodevelopmental Outcomes Associated with Early-Life Exposure to Heavy Metals: A Systematic Review
Source: Int J Environ Res Public Health. 2025 Aug 21;22(8):1308. doi: 10.3390/ijerph22081308 (PMC12386800; doi:10.3390/ijerph22081308)
Supplement: Supplementary file 1 [file ijerph-22-01308-s001.zip › Supplementary material - Table S2.pdf]

Neurodevelopmental Outcomes Associated with Early-Life Exposure to Heavy Metals: A Systematic Review

Supplementary material - Table S2

Table S2: Descriptive characteristics of the include studies

AUTHOR / YEAR / COUNTRY / TYPE OF STUDY	N | AGE | GENDER % | PURPOSE	METAL ANALYSIS	NEURODEVELOPMENTAL ASSESSMENT	MAJOR ASSOCIATIONS	Quality
Abd Wahil et al., 2022<br><br>Malaysia<br><br>Cross-sectional	Typically Develop: 74<br><br>Age: 5.45 (0.83) years<br><br>Males: 39 (52.70%) males<br><br>ASD: 81<br><br>Age: 5.63 (0.60) years<br><br>Males: 68 (84%) males<br><br>To assess the role of Pb and essential trace elements in the development of ASD among preschool children	Exposure: Not informed<br><br>Sample: Child urine<br><br>Method: Inductively coupled plasma mass spectrometry (ICP-MS)<br><br>Metal: Pb	ASD diagnoses by DSM-5 criteria and International Classification of Diseases-10 (ICD-10)	Urinary Pb levels were significantly lower in children with ASD (mean 0.26 ± 0.31 μg/dL) compared to typically developing children (mean 0.58 ± 0.41 μg/dL) ($p < 0.05$).	***
Al-Saleh(a) et al., 2016<br><br>Saudi Arabia<br><br>Cross-sectional	N: 944<br><br>Age: 7.96 (2.68) months<br><br>Gender: 487 (51.6%) males<br><br>To evaluate postnatal exposure to different forms of organic and inorganic Hg and their association with delayed neurological development.	Exposure: Not informed<br><br>Sample: Maternal blood, milk and urine / Child hair, blood and urine.<br><br>Method: Varian FS-240 Atomic Absorption Spectrophotometer<br><br>Metal: Hg	Denver Developmental Screening Test II (DDST-II)<br><br>Parents' Evaluation of Developmental Status (PEDS)	Neonatal hair and urinary Hg levels were positively associated with an increased risk of future learning problems (OR = 1.209, 95% CI = 1.001–1.460).<br>Neonatal hair MeHg levels were positively associated with potential delays in overall development (OR = 1.193, 95% CI = 1.044–1.365).<br>Neonatal hair and urinary Hg levels were positively associated with an increased risk of future behavioral problems by Parents Evaluation of Developmental Status scale (OR = 1.337, 95% CI = 1.012–1.767).	***
Al-Saleh(b) et al., 2016<br><br>Saudi Arabia<br><br>Cross-sectional	N: 944<br><br>Idade: 8.0 (2.7) months<br><br>Gender: 487 (51.6%) males<br><br>To analyze changes in biochemical markers associated with Hg	Exposure: Not informed<br><br>Sample: Maternal and child Urine and hair / Maternal blood and milk<br><br>Method: Varian FS-240 Atomic	Denver Developmental Screening Test & II (DDST and DDST-II)<br><br>Parents' Evaluation of Developmental Status (PEDS)	Maternal hair MeHg levels were positively associated with delayed development risk test outcomes (adjusted OR = 1.193, p = 0.01).	***

	exposure and their relationship with child neurodevelopment.	Absorption Spectrophotometer<br><br>Metal: Hg			
Al-Saleh et al., 2020<br><br>Longitudinal<br><br>Saudi Arabia	N: 82<br><br>Age: 6.81 (0.64) years<br><br>Gender: 36 (43.9%) males<br><br>To investigate the impact of early (during breastfeeding) and recent (during follow-up) exposure to Hg and Pb on the neurodevelopment of children aged 5 to 8 years.	Exposure: Not informed<br><br>Sample: Maternal blood, hair and urine / Child hair and urine<br><br>Method: Varian FS-240 Atomic Absorption Spectrophotometer and Varian AA-240 Zeeman atomic absorption spectrophotometer coupled to a GTA-120 electrothermal atomizer<br><br>Metal: Pb and Hg	Beery-Visual-Motor Integration (Beery VMI)<br><br>Test Of Non-Verbal Intelligence (TONI)	Urinary Hg levels in infants during breastfeeding were associated with lower visual-motor integration (VMI) scores (ß = −0.469, 95% CI = −7.541, −2.13).<br>Urinary Hg levels in infants during breastfeeding were associated with lower non-verbal intelligence scores (ß = −0.359, 95% CI = −5.857, −0.845).<br>No correlation was found between neurodevelopmental scores and levels of MeHg in hair, Hg in urine, or Pb in urine among children during follow-up.	**
Boucher et al., 2010<br><br>Canada<br><br>Longitudinal	N: 118<br><br>Age: 11.3 (0.57) years<br><br>Gender: 41 (34.7%) males<br><br>To examine the association between prenatal and current exposures to MeHg and PCBs and event-related potential measures of information processing during an auditory oddball task in a cohort of Inuit children from Arctic Quebec exposed to these contaminants through the consumption of marine mammals and fish.	Exposure: Marine mammal and fish consumption<br><br>Sample: Umbilical cord and venous blood<br><br>Method: Cold vapor atomic absorption spectrometry and graphite furnace atomic absorption with Zeeman background correction<br><br>Metal: MeHg	Event-related potentials (ERPs)<br><br>Electro-oculogram (EOG)	Umbilical cord blood MeHg concentrations were not associated with children's neurodevelopmental parameters, in the follow-up	***
Cai et al., 2019<br><br>China<br><br>Cross-sectional	N: 574<br><br>Age: 3-6 years<br><br>Gender: 305 (53.13%) males<br><br>To examine the effects of Pb exposure in children living in areas contaminated by electronic waste and its association with sensory	Exposure: Electronic waste zones<br><br>Sample: Child blood<br><br>Method: Graphite furnace atomic absorption spectrophotometry<br><br>Metal: Pb	Sensory Processing Measure-Hong Kong Chinese version (SPM-HKC)	Children’s blood Pb levels were positively associated with the total score of sensory processing dysfunction ($r = 0.168$, $p < 0.01$).	***

	integration difficulties, as well as to investigate the role of the HPA axis in modulating these difficulties.				
de Assis Araújo et al., 2022<br><br>Brazil<br><br>Longitudinal	N: 48<br><br>Age: At birth - 6 months<br><br>Gender: 31 (62%) males<br><br>To investigate the association between metal concentrations in maternal and umbilical cord blood and the neurological development of newborns from the pilot population of the PIPA study.	Exposure: Not informed<br><br>Sample: Maternal and umbilical cord blood<br><br>Method: Inductively Coupled Plasma Mass Spectrometry technique (ICP-MS)<br><br>Metal: As, Cd, Pb and Hg	Denver Developmental Screening Test II (DDST-II)	Maternal blood As levels were higher in the group of children with more failures on the global neurodevelopmental test ($p = 0.03$).	**
Deroma et al., 2013<br><br>Italy<br><br>Longitudinal	N: 154<br><br>Age: 7.7 (0.7) years<br><br>Gender: 77 (50%) males<br><br>To assess the impact of prenatal and postnatal low-dose Hg exposure and fish consumption on the intelligence quotient (IQ) of school-aged children, considering the potential beneficial effects of fish and other confounding factors.	Exposure: Soil and water pollution<br><br>Sample: Maternal hair and milk / Child hair<br><br>Method: Gold amalgamation cold vapour atomic absorption spectrometry<br><br>Metal: Hg	Wechsler Intelligence Scale for Children (WISC-III)	Total Hg or MeHg levels in hair were not associated with children's IQ scores.	***
Desrochers-Couture et al., 2018<br><br>Canada<br><br>Longitudinal	N: 609<br><br>Age: 3.4 (0.3) years<br><br>Gender: 297 (48.8%) males<br><br>To examine the association between blood Pb concentrations and cognitive function in Canadian preschool children, considering a potential moderating effect of sex.	Exposure: Not informed<br><br>Sample: Maternal and umbilical cord blood<br><br>Method: Inductively coupled plasma mass spectrometry<br><br>Metal: Pb	Wechsler Preschool and Primary Scale of Intelligence (WPPSI-III)	Cord blood Pb levels were negatively correlated with IQ in boys ($b = -3.28$).<br>Umbilical cord blood Pb levels showed no significant correlation with Performance IQ in girls ($\beta = -3.28$).	***
Farías et al., 2022	N: 253<br><br>Age: 1-12 months	Exposure: Not informed	Bayley Scales of Infant Development (BSID-III)	Maternal blood Pb levels were negatively associated with offspring language development ($p < 0.05$).	**

Mexico<br><br>Longitudinal	Gender: 132 (52.1%) males<br><br>To study the associations between prenatal levels of lPb, Hg, and Mn, both jointly and separately, and neurodevelopment during the first year of life.	Sample: Maternal blood<br><br>Method: Atomic absorption spectrophotometry<br><br>Metal: Pb			
Freire et al., 2010<br><br>Spain<br><br>Cross-sectional	N: 72<br><br>Age: 51 months<br><br>Gender: 72 (100%) males<br><br>To investigate Hgexposure in preschool children from Granada, Spain, by quantifying total Hg (T-Hg) levels in hair, to examine the influence of fish consumption during childhood and other factors on T-Hg exposure, and to assess their combined effects on cognitive and motor development at 4 years of age.	Exposure: Water and fish consumption<br><br>Sample: Child hair<br><br>Method: Gold amalgamation, thermal desorption and atomic absorption spectrometry<br><br>Metal: Hg	McCarthy Scales of Children's Abilities (MSCA)<br><br>General Cognitive Score (GCS)	Children's total hair Hg levels (T-Hg in µg/g) showed negative associations with neurodevelopment at 4 years of age, specifically in the domain of general cognition (β = −2.09, 95% CI = −5.72 to 1.54). Children's total hair Hg levels (T-Hg in µg/g) showed negative associations with neurodevelopment at 4 years of age in the domains of gross motor skills (β = −1.09, 95% CI: −5.25 to 3.07) and fine motor skills (β = −1.03, 95% CI: −5.46 to 3.41).	**
Guo et al, 2020.<br><br>China<br><br>Longitudinal	N: 326<br><br>Age: 7.4 (0.4) years<br><br>Gender: 186 (57%) males<br><br>To investigate the association between prenatal exposure to heavy metals and intellectual development in 7-year-old children.	Exposure: Agriculture and pesticides<br><br>Sample: Maternal urine<br><br>Method: Inductively Coupled Plasma Mass Spectrometry (ICP-MS)<br><br>Metal: Cd, Pb and Hg	Chinese Revised-Wechsler Intelligence Scale for Children (C-WISC-IV)<br><br>Verbal Intelligence Quotient (VIQ)<br><br>Performance Intelligence Quotient (PIQ)<br><br>Full Intelligence Quotient (FIQ),	Prenatal maternal urinary Pb levels were negatively associated with children's total IQ at 7 years of age (β = −2.31, 95% CI: −4.13 to −0.48; p = 0.013).	**
Halabicky et al., 2023<br><br>China<br><br>Longitudinal	N: 417<br><br>Age: 11.51 (0.39) years<br><br>Gender: 220 (52.76%) males<br><br>To examine the combined and interactive effects of childhood blood Pb levels (BLLs) and parental education on	Exposure: Not informed<br><br>Sample: Child blood<br><br>Method: Atomic absorption spectrophotometer with graphite furnace<br><br>Metal: Pb	Wechsler Intelligence Scale for Children-Revised (WISC-R)<br><br>Working Memory Measurement Software (WM)	Children's blood Pb levels were associated with lower IQ scores (β = −0.59) at 3 to 5 years of age.<br><br>No association was found between Pb blood levels and worsened IQ outcomes (β = −0.59) in 12-year-old children.	**

	neurocognition in early adolescence.				
Hu et al., 2006<br><br>Mexico<br><br>Longitudinal	N: 146<br><br>Age: Not applicate<br><br>Gender: 76 (52.05%) males<br><br>To study the impact of prenatal Pb exposure on neurodevelopment using repeated measures of fetal dose as reflected by maternal whole blood and plasma Pb levels.	Exposure: Air pollution from burning gasoline containing Pb<br><br>Sample: Umbilical cord and venous blood<br><br>Method: Atomic Absorption Spectrometry (AAS)<br><br>Metal: Pb	Bayley Scales of Infant Development II–Spanish version (BSID-IIS)	Increased plasma Pb and whole blood Pb levels during the first trimester of pregnancy were associated with lower mental development scores by BSID-II scale at 24 months of age (β = -3.54, p = 0.03 and β = -2.4, p = 0.19, respectively).	***
Hu et al., 2016<br><br>China<br><br>Cross-sectional	N: 410<br><br>Age: Not applicable<br><br>Gender: 214 (52.19%) males<br><br>To assess Hg levels in neonates and their mothers, examine whether low prenatal Hg exposure affects neurodevelopment in 1-year-old children, and analyze the potential confounding role of fish consumption on Hg exposure and developmental outcomes in infants.	Exposure: Water and fish consumption<br><br>Sample: Maternal and umbilical cord blood<br><br>Method: Cold Vapor Atomic Absorption Spectrometry (AAS)<br><br>Metal: Hg	Gesell Developmental Schedules (GDS)	Umbilical cord blood Hg levels were positively associated with developmental quotients in the adaptive domain by GDS scale (β = 4.22) and the social domain (β = 4.06).	***
Huang et al., 2012<br><br>Taiwan<br><br>Longitudinal	N: 119<br><br>Age: 2–9 years<br><br>Gender: 62 (52.1%) males<br><br>To investigate the effects of prenatal and postnatal exposure to low levels of Pb on cognitive development in children over a 9-year period following the ban on leaded gasoline in Taiwan.	Exposure: Air pollution from burning gasoline containing Pb<br><br>Sample: Umbilical cord and venous blood<br><br>Method: Zeeman effect graphite furnace atomic absorption spectrometry (GF-AAS)<br><br>Metal: Pb	Bayley Scales of Infant Development-II (BSID-II)<br><br>Wechsler Preschool and Primary Scale of Intelligence-Revised (WPPSI-R)<br><br>Wechsler Intelligence Scale for Children-version III (WISC-III)	Children's blood Pb levels were negatively associated with total IQ scores between ages 5 and 6 years (β = −5.97, SE = 2.59, p = 0.025) and between ages 2 and 9 years (β = −0.289, 95% CI: −16.9 to −1.48, p = 0.020).	***

Inoue et al., 2022<br><br>Japan<br><br>Longitudinal	N: 80759<br><br>Age: Not applicable<br><br>Gender: 41,190 (51%) males<br><br>To investigate the association between prenatal blood Pb levels and delays in neurodevelopment during the first three years of life using a national cohort.	Exposure: Not informed<br><br>Sample: Maternal and umbilical cord blood<br><br>Method: Inductively Coupled Plasma Mass Spectrometry (ICP-MS)<br><br>Metal: Pb	Ages and Stages Questionnaires (ASQ-III)	No significant associations were found between Pb levels in umbilical cord blood and suspected neurodevelopmental delay during the first three years of life.	**
Kao et al., 2021<br><br>Taiwan<br><br>Cross-sectional	N: 139<br><br>Age: 2.8 (0.4) years<br><br>Gender: 66 (47%) males<br><br>To explore links between land use patterns and children's exposure to soil lead, using hair and nail Pb levels as biomarkers	Exposure: Contaminated soil<br><br>Sample: Child hair and nail<br><br>Method: Inductively coupled plasma and mass spectrometer<br><br>Metal: Pb	Bayley Scales of Infant Development-III (BSID-III)	Children's hair Pb concentrations were negatively associated with gross motor scores (β = −0.04, 95% CI: −0.07 to −0.01) after adjustment for residence near a highway.	***
Kao et al., 2023<br><br>Taiwan<br><br>Longitudinal	N: 152<br><br>Age: At 24 months<br><br>Gender: 72 (47.36%) males<br><br>To investigate the association between childhood exposure to multiple metals and low birth weight in preterm infants, and its impact on neurodevelopment at 24 months.	Exposure: Not informed<br><br>Sample: Child hair and nail<br><br>Method: Inductively coupled plasma-mass spectrometry (ICP-MS)<br><br>Metal: Pb, As, Cd, and Se	Bayley Scales of Infant Development-III (BSID-III)	In low birth weight premature children, Cd concentrations in the nails were negatively associated with cognition (β = −0.63, 95% CI: −1.17 to −0.08).	***
Kashala-Abotnes et al., 2016<br><br>Democratic Republic of Congo<br><br>Cross-sectional	N: 89<br><br>Age: 17.5 (4.3) months<br><br>Gender: 52 (58.4%) males<br><br>To determine the association between Pb exposure and neurodevelopment and behavior in children aged 12 to 24 months.	Exposure: Kitchen utensils made of Pb<br><br>Sample: Child blood<br><br>Method: Flame atomic absorption spectrophotometry<br><br>Metal: Pb	Baby characteristics questionnaire (BCQ)	No statistically significant differences were observed in child neurodevelopment.	***

Kim et al., 2009<br>South Korea<br>Cross-sectional	N: 261<br>Age: 9.7 (0.6) years<br>Gender: 141 (54%) males<br>To investigate the association between environmental levels of Pb and Mn and intellectual function in school-aged children, as well as to explore the interactions of concurrent exposure to these two heavy metals on intellectual function.	Exposure: Not informed<br>Sample: Venous blood<br>Method: Atomic absorption spectrometer with a graphite furnace<br>Metal: Pb and Mn	Korean Educational Development Institute-Wechsler Intelligence Scales (KEDI-WISC)	Pb levels were negatively associated with total IQ (β = −0.174) and verbal IQ (β = −0.187).	**
Kim et al., 2018<br>South Korea<br>Longitudinal	N: 1098<br>Age: 6-36 months<br>Gender: 571 (52%) males<br>To investigate the influence of prenatal Hg exposure, adjusted for maternal fish consumption frequency on neurocognitive development	Exposure: Water and fish consumption<br>Sample: Maternal and umbilical cord blood<br>Method: Gold amalgam mercury analyzer.<br>Metal: Hg	Bayley Scales of Infant Development-II (BSID-II) to evaluate the Psychomotor Development Index (PDI) and Mental Development Index (MDI)	Prenatal exposure to Hg in early pregnancy was inversely associated with psychomotor development index (PDI) scores at 6 months (β = −0.550, p = 0.031).<br>Prenatal exposure to Hg in early pregnancy was inversely associated with mental development index (MDI) scores at 6 months (β = −0.408, p = 0.048).	***
Kippler et al., 2016<br>Greece<br>Longitudinal	N: 575<br>Age: 4.2 (0.23) years<br>Gender: 288 (50%) males<br>To evaluate the impact of prenatal exposure to heavy metals on neurodevelopment at 4 years of age.	Exposure: Food<br>Sample: Maternal urine<br>Method: Inductively Coupled Plasma Mass Spectrometry (ICPMS)<br>Metal: Cd, Se and I.	McCarthy Scales of Children's Abilities (MSCA)	Maternal urinary Cd concentrations ≥0.8 µg/L during pregnancy were inversely associated with children's overall cognitive scores at 4 years of age (β = −6.2, 95% CI: −12 to −0.54, p = 0.032).<br>Maternal urinary Pb concentrations ≥0.8 µg/L during pregnancy were not associated with children's overall cognitive scores (β = −6.2; 95% CI: −12, 0.54; p = 0.45).	***
Kou et al., 2025<br>Spain<br>Longitudinal	N: 400<br>Age: 40 days<br>Gender: 210 (52.5%) males	Exposure: Not informed<br>Sample: Maternal urine<br>Method: Inductively Coupled Plasma	Bayley Scales of Infant and Toddler Development-III (BSID-III)	The mixture of heavy metals: Cd, Ni, Hg, and Pb was associated with poorer expressive language scores (β = −0.26, 95% CI: −0.44 to −0.07). Cd was the most influential contributor to lower cognitive scores (β = −1.47, p = 0.044).	***

	To investigate the combined effect of prenatal exposure to heavy metal on child neurodevelopment.	Tandem Mass Spectrometry (ICP-MS)<br><br>Metal: Cd, Ni, Hg and Pb.			
Lee et al., 2017<br><br>South Korea<br><br>Longitudinal	N: 251<br><br>Age: 6-60 months<br><br>Gender: Not informed<br><br>To evaluate the association between heavy metal levels in umbilical cord blood and cognitive development stability.	Exposure: Not informed<br><br>Sample: Umbilical cord blood<br><br>Method: Atomic absorption spectrometry and gold amalgamation direct mercury analyzer<br><br>Metal: Cd, Pb and Hg	The Korean version of Bayley Scales of Infant and Toddler Development-II (K-BSID-II) to evaluate the Psychomotor Development Index (PDI) and Mental Development Index (MDI)<br><br>The Korean version of the Wechsler Preschool and Primary Scale of Intelligence – Revised (K-WPPSI-R)	Cognitive development stability appeared to be unaffected by heavy metal levels in umbilical cord blood.	**
Lee et al., 2018<br><br>Taiwan<br><br>Cross-sectional	N:122<br>Health control:<br>N: 46<br>Age: 8.1 (1.2) years<br>Gender: 31 (67.4%) males<br>ADHD-I :N: 29<br>Age: 8.0 (1) years<br>Gender: 11 (37.93%) males<br>TDAH-H/I: N: 47<br>Age: 7.7 (1) years<br>Gender: 40 (85.10%) males<br><br>To explore the relationships between various heavy metals, neurocognitive function, and ADHD symptoms.	Exposure: Not informed<br><br>Sample: Child urine<br><br>Method: Inductively Coupled Plasma Mass Spectrometry (ICP-MS)<br><br>Metal: Mn, Pb, Cd, Hg, Bi and Sb	Schedule for Affective Disorders and Schizophrenia for School-Age Children, epidemiologic version (K-SADS-E)<br><br>Wechsler Intelligence Scale for Children–Fourth Edition (WISC-IV)<br><br>Swanson, Nolan, and Pelham Version IV Scale (SNAP-IV)	Cd ($p < 0.05$) and Pb ($p < 0.01$) levels were negatively correlated with the IQ.<br>Sb ($p < 0.01$) and Pb ($p < 0.05$) levels were positively correlated with the severity of ADHD symptoms	**
Lee et al., 2021<br><br>South Korea<br><br>Longitudinal	N: 502<br><br>Age: 4-6 years<br><br>Gender: 254 (50.6%) males<br><br>To investigate the association between prenatal and postnatal metal exposure at ages 4 and 6 and children's IQ measured at age 6.	Exposure: Not informed<br><br>Sample: Maternal and child blood<br><br>Method: Atomic absorption spectrophotometry<br><br>Metal: Mn, Hg, Cd and Pb.	Korean Educational Developmental Institute's Wechsler Intelligence Scale for Children (KEDI-WISC)	Blood Mn levels at 4 years of age were negatively associated with children's IQ ($\beta = -5.99$, 95% CI: −11.37 to −0.61).	***

Lin et al., 2013<br><br>Taiwan<br><br>Cross-sectional	N: 230<br><br>Age: At birth to 2 years<br><br>Gender: 128 (55.7%) males<br><br>To explore the relationship between in utero exposure to environmental neurotoxic metals and neurodevelopment at 2 years of age.	Exposure: Not informed<br><br>Sample: Umbilical cord blood<br><br>Method: Inductively Coupled Plasma Mass Spectrometry (ICP-MS)<br><br>Metal: Mn, Pb, As and Hg.	Comprehensive developmental inventory for infants and toddlers (CDIIT)	General developmental quotients were significantly lower in the group with high exposure to Mn and Pb (β = −7.03, SE = 2.65, p = 0.009).<br>High exposure to Mn and Pb was associated with lower cognitive quotients (β = −8.19, SE = 3.17, p = 0.011) and language quotients (β = −6.81, SE = 2.73, p = 0.013).<br>No significant differences were found in language development quotients (β = −6.81, SE = 2.73, p = 0.013) in the group with high exposure to As or Hg.<br>No significant differences were found in cognitive development quotients (β = −8.19, SE = 3.17, p = 0.011) in the group with high exposure to As or Hg.	***
Liu et al., 2024<br><br>Mexico<br><br>Longitudinal	N: 533<br><br>Age: 9 (9-10) years<br><br>Gender: 274 (51.4%) males<br><br>To model inhibitory control using item response theory across tasks and introduce a resampling method to address measurement error in its association with Pb exposure.	Exposure: Not informed<br><br>Sample: Umbilical cord blood<br><br>Method: Agilent 8800 ICP Triple Quad (ICP-QQQ)<br><br>Metal: Pb	Go/NoGo Happy, Go/NoGo Neutral, Go/NoGo Letter and D-KEFS Color Word Interference Test	Pb concentrations in umbilical cord blood (β = −0.06, 95% CI: −0.11 to −0.01) and in blood at 4 years of age (β = −0.07, 95% CI: −0.12 to −0.02) were negatively associated with inhibitory control in childhood. There was a significant negative association between umbilical cord blood Pb levels and childhood hyperactivity (β = −0.117, p = 0.009).	**
Liu J. (a) et al., 2014<br><br>China<br><br>Longitudinal	N: 243<br><br>Age: 6-36 months<br><br>Gender: 129 (53.09%) males<br><br>To investigate the adverse cognitive effects of low-level Pb exposure during the prenatal and postnatal periods, monitored over the first 3 years of life in the Pearl River Delta region, China.	Exposure: Not informed<br><br>Sample: Umbilical cord blood and venous blood<br><br>Method: Atomic absorption spectrophotometry with graphite furnace<br><br>Metal: Pb	Bayley Scales of Infant Development-II (BSID-II) to evaluate the Psychomotor Development Index (PDI) and Mental Development Index (MDI)	Significant deficit in psychomotor development index (PDI) due to prenatal Pb exposure at 36 months (β = −1.302, p = 0.041).<br>Umbilical cord blood Pb levels were inversely associated with mental development index scores (β = −1.291, p = 0.036).	***
Liu J. (b) et al., 2014	N: 332<br><br>Age: 3 days	Exposure: Air pollution, paint, hair dye and cosmetics	Neonatal behavioral neurological assessments (NBNA)	Infant exposure to Pb during the first trimester of pregnancy was positively associated with reduced neonatal development scores (β =	***

China<br><br>Longitudinal	Gender: 185 (55.72%) males<br><br>To determine neurotoxicity during specific trimesters, analyze "safe" levels of Pb exposure, and identify factors influencing prenatal Pb exposure.	Sample: Maternal blood<br><br>Method: Atomic absorption spectrophotometry with graphite furnace.<br><br>Metal: Pb		−4.86, 95% CI: −8.831 to −0.889, p = 0.03).	
Llop et al., 2012<br><br>Spain<br><br>Longitudinal	N: 1.683<br><br>Age: 14 (11-23) months<br><br>Gender: 882 (52.4%) males<br><br>To evaluate whether Hg levels in umbilical cord blood affect mental and psychomotor development in infants from areas with moderate to high fish consumption.	Exposure: Water and fish consumption<br><br>Sample: Umbilical cord blood<br><br>Method: Atomic absorption spectrometry<br><br>Metal: Hg	Bayley Scales of Infant Development	No overall significant association was found between Hg levels and neurodevelopment.	***
Llop et al., 2016<br><br>Spain<br><br>Cross-sectional	N: 1362<br><br>Age: 4.8 (0.61) years<br><br>Gender: 712 (52.3%) males<br><br>To assess the association between prenatal Hg exposure and neuropsychological development in childhood.	Exposure: Water and fish consumption<br><br>Sample: Umbilical cord blood<br><br>Method: Atomic absorption spectrometry<br><br>Metal: Hg	McCarthy Scales of Children's Abilities (MSCA)	Umbilical cord blood Hg concentrations were positively associated with cognitive development scale scores (β = 1.29, 95% CI: 0.28 to 2.31).	***
Lu et al., 2023<br><br>China<br><br>Longitudinal	N: 275<br><br>Age: 18.32 (0.68) months<br><br>Gender: 140 (50.91) males<br><br>To further explore the relationship between prenatal Pb exposure and motor, language, and cognitive development in infants.	Exposure: Not informed<br><br>Sample: Umbilical cord blood<br><br>Method: Atomic absorption spectrometry<br><br>Metal: Pb	Bayley Scales of Infant and Toddler Development-III (BSID-III)	Umbilical cord blood Pb levels were associated with lower fine motor control scores in girls (β = −1.5, 95% CI: −2.6 to −0.4).	**
Ma et al., 2021<br><br>Japan	N: 3545<br><br>Age: At birth to 2 years	Exposure: Not informed	Kyoto Scale of Psychological Development (KSPD)	Elevated Cd concentrations in maternal or umbilical cord blood were not significantly associated with neurodevelopmental delay.	***

Longitudinal	Gender: 1781 (50.2%) males<br><br>To investigate associations between prenatal Cd exposure and neurodevelopment in 2-year-old children, and the influence of maternal and child characteristics.	Sample: Umbilical cord blood and maternal blood<br><br>Method: Inductively Coupled Plasma Mass Spectrometry (ICP-MS)<br><br>Metal: Cd			
Marques et al., 2011<br><br>Brazil<br><br>Cross-sectional	N: 668<br><br>Age: 19.82 (14.47) months<br><br>Gender: Not informed<br><br>To assess linear growth and neurodevelopment in children under 5 years of age living in open-pit mining environments, in relation to family fish consumption.	Exposure: Water, fish consumption and mining area<br><br>Sample: Child hair<br><br>Method: Cold vapor atomic absorption spectrometry<br><br>Metal: Hg	Gesell Developmental Scores (GDS)	Children's average Hg concentration was positively correlated with neurodevelopment scores by GDS scale (r = 0.080, p = 0.035).	**
Marques et al., 2014<br><br>Brazil<br><br>Longitudinal	N: 294<br><br>Age: 6-24 months<br><br>Gender: 105 (35.71%) males<br><br>To identify potential associations between neurobehavioral test outcomes and Hg exposure in an open-pit tin mining environment in Brazil.	Exposure: Water, land and mining area<br><br>Sample: Child hair<br><br>Method: Cold-vapor atomic absorption spectrometry<br><br>Metal: Hg	Bayley Scales of Infant and Toddler Development-II (BSID-II) to evaluate the Psychomotor Development Index (PDI) and Mental Development Index (MDI)	Neonatal hair Hg levels showed a significant negative association with mental development index scores in boys at 24 months (β = −0.222, 95% CI: −0.44 to −0.01, p = 0.0451). Neonatal hair Hg levels showed a positive association with age at walking onset in girls (β = 0.188, 95% CI: 0.032 to 0.344, p = 0.019).	**
Masumoto et al., 2022<br><br>Japan<br><br>Longitudinal	N: 96165<br><br>Age: At birth to 3 years<br><br>Gender: 49257 (51,2%) males<br><br>To investigate the relationship between maternal blood Cd concentration during pregnancy and child development.	Exposure: Not informed<br><br>Sample: Maternal blood<br><br>Method: Inductively coupled plasma mass spectrometry<br><br>Metal: Cd	Ages and Stages questionnaires (ASQ-3)	Blood Cd concentration was associated with gross motor function delay 1.5 years after birth (adjusted OR = 1.19, 99.7% CI: 1.01–1.40).	***
Merced-Nieves et. al., 2022	N: 549	Exposure: Not informed	National Center for Toxicological Research (NCTR)	Umbilical cord blood Pb levels were associated with altered temporal perception in children (p = 0.02).	**

Mexico<br><br>Longitudinal	Age: 6.7 (0.5) years<br><br>Gender: 278 (50.6%) males<br><br>To assess the association between Pb exposure and cognitive development deficits, identifying the underlying mechanisms. A battery of tests was used, and effect modification by child sex was evaluated.	Sample: Maternal blood and umbilical cord blood<br><br>Method: Agilent 8800 ICP Triple Quad (ICP-QQQ)<br><br>Metal: Pb	Operant Test Battery (OTB)		
Myers et al., 2004<br><br>Seychelles<br><br>Longitudinal	N: 643<br><br>Age: 8.97 (0.33) years<br><br>Gender: Not informed<br><br>To examine the association between MeHg exposure and scores on the ten subscales of the CBCL administered at 107 months	Exposure: Not informed<br><br>Sample: Maternal and child hair<br><br>Method: Cold vapour atomic absorption<br><br>Metal: Hg	Child Behaviour Checklist (CBCL), WISC	There was a positive association between postnatal Hg exposure and Thought Problems ($p = 0.011$).There was a significant negative association between prenatal Hg exposure and Social Problems scores ($\beta = -0.029$).	**
Naspolini et al., 2024<br><br>Brazil<br><br>Longitudinal	N: 157<br><br>Age: 3-16 months<br><br>Gender: 76 (48.41) males<br><br>To investigate the association between infants' exposure to As, Pb, Hg, and Cd through human milk and adverse neurodevelopmental outcomes.	Exposure: Not informed<br><br>Sample: Breast milk<br><br>Method: Inductively Coupled Plasma Mass Spectrometry (ICP-MS)<br><br>Metal: As, Cd, Pb and Hg.	Bayley Scales of Infant and Toddler Development-III (Bayley-III)	Infants exposed to Pb showed significantly lower language performance at 10–16 months of age ($\beta = -0.413$; 95% CI: −0.653 to −0.173) compared to unexposed infants.	*
Nie et al., 2012<br><br>USA<br><br>Cross-sectional	N: 11<br><br>Age: 11 (1.6) years<br><br>Gender: 55% males<br><br>To identify the best marker for assessing Pb exposure in children.	Exposure: Not informed<br><br>Sample: Blood<br><br>Method: Inductively coupled plasma-mass spectrometry (ICP-MS)<br><br>Metal: Pb	Wechsler Intelligence Scale for Children–Fourth Edition (WISC-IV)<br><br>Behavior Rating Inventory of Executive Function (BRIEF)<br><br>Conners ADHD/DSM-IV Scale (CADS-IV)	Blood Pb levels were associated with increased externalizing problems, internalizing problems, and behavioral symptoms ($r = 0.943$, $r = 0.648$, and $r = 0.853$, respectively). Blood Pb levels showed a positive association with Verbal Comprehension, Working Memory, and IQ ($r = 0.746$, $r = 0.853$, and $r = 0.823$, respectively).	***

Notario-Barandiaran et al., 2024<br><br>Spain<br><br>Cross-sectional	N: 962<br><br>Age: 4.45 (4-6.4) years<br><br>Gender: 502 (52.2%) males<br><br>To identify metal mixture patterns and assess their impact on child neurodevelopment.	Exposure: Not informed<br><br>Sample: Urine<br><br>Method: Inductively coupled plasma mass spectrometry (ICP-MS)<br><br>Metal: Co, Cu, Mo, Se, Pb, Zn and As	McCarthy Scales of Children's Abilities (MSCA)	Exposure to the mixture of Cu Se, Pb, and Zn was associated with poorer verbal executive function (β = −1.88, 95% CI: −3.17 to −0.59). Exposure to the mixture of inorganic and organic As was associated with poorer gross motor function (β = −1.41, 95% CI: −2.36 to −0.46).	***
Nozadi et al., 2021<br><br>USA<br><br>Cross-sectional	N: 327<br><br>Age: 10-13 months<br><br>Gender: 163 (49.8%) males<br><br>To overcome the limitations of existing analyses of individual metals and clusters by using a statistical causal inference technique along with a priori causal structures to estimate the effects of a single metal on health outcomes, controlling for potential residual confounding effects due to co-exposed metals.	Exposure: Abandoned uranium mine region<br><br>Sample: Maternal urine and blood<br><br>Method: Inductively coupled plasma-dynamic reaction cell-mass spectrometry (ICP-DRC-MS)<br><br>Metal: As, Ba, Be, Cd, Co, Cs, I, Pb, Mn, Hg, Mo, Pt, Sr, Sn, Tl, W and U.	Ages and Stages Questionnaire Inventory (ASQ:I)	Problem−solving ability showed negative correlations with urinary Mo, Sb, and As levels (r = −0.001, r = −0.106, and r = −0.124, respectively). Fine motor skills showed negative correlations with urinary Ba and As levels (r = −0.132 and r = −0.156, respectively). Social behavior showed negative correlations with urinary As and urinary U levels (r = −0.100 and r = −0.01, respectively). Fine motor skills showed positive correlations with blood Cd, urinary Cs, urinary Mo, urinary Sr, and urinary W levels (r = 0.070, r = 0.038, r = 0.065, r = 0.021, and r = 0.120, respectively). Gross motor skills showed a positive correlation with urinary Sr levels (r = 0.113). Urinary W levels were positively correlated with communication development (r = 0.136). Problem−solving ability showed positive correlations with urinary Cs, Sn, Sr, Tl, and W levels (r = 0.039, r = 0.073, r = 0.089, r = 0.120, and r = 0.079, respectively).	**
Nyanza et al., 2021	N: 439	Exposure: Artisanal gold mining area	Malawi Developmental	Higher prenatal As exposure was positively associated with an increased risk of social impairment in girls (adjusted prevalence ratio [aPR] = 1.01, 95% CI: 1.00–1.02, p	**

Tanzania<br><br>Longitudinal	Age: 7.92 (1.77) months<br><br>Gedner: 213 (48.6%) males<br><br>To examine the associations between prenatal exposure and co-exposure to Pb, Hg, Cd, and As and child neurodevelopment outcomes in areas with artisanal and small-scale gold mining (ASGM) activities in Tanzania.	Sample: Maternal blood and urine<br><br>Method: Inductively coupled plasma mass spectrometry (ICP-MS)<br><br>Metal: As, Cd, Pb and Hg	Assessment Tool (MDAT)	< 0.05). Higher prenatal Hg exposure was positively associated with global neurodevelopmental impairment in girls (adjusted prevalence ratio [aPR] = 1.10, 95% CI: 1.06–1.14, $p < 0.001$). Prenatal exposure to As and Cd was positively associated with global neurodevelopmental impairment in boys (adjusted prevalence ratios [aPR] = 1.03, 95% CI: 1.02–1.048, $p < 0.001$; and aPR = 2.55, 95% CI: 1.33–4.87, $p < 0.05$, respectively). Higher prenatal Hg exposure was positively associated with language impairment in boys (adjusted prevalence ratio [aPR] = 1.05, 95% CI: 1.04–1.07) and girls (aPR = 1.21, 95% CI: 1.16–1.27, $p < 0.001$).	
Orenstein et al., 2014<br><br>USA<br><br>Cross-sectional	N: 393<br><br>Age: 8.1 (0.6) years<br><br>Gender: 196 (49.9%) males<br><br>To assess the association of low-level joint prenatal exposures to organochlorines and MeHg.	Exposure: Industrial waste, water and land<br><br>Sample: Maternal hair<br><br>Method: Atomic absorption<br><br>Metal: Hg	Wide Range Assessment of Memory and Learning (WRAML)	The figure memorization capacity index (visual memory) was negatively associated with MeHg concentration ($\beta = -3.1$).	***
Parajuli et al., 2013<br><br>Nepal<br><br>Cross-sectional	N = 100<br><br>Age: 17.4 (3.3) hours<br><br>Gender: Not informed<br><br>To investigate the impact of in utero toxic element exposures and levels of the essential element Zn on neurodevelopmental indicators at birth.	Exposure: Highway air and water pollution<br><br>Sample: Umbilical cord blood<br><br>Method: inductively coupled plasma mass spectrometer<br><br>Metal: As and Pb	Brazelton neonatal behavioral assessment scale (NBAS III)	Pb and As levels in umbilical cord blood were negatively associated with motor function scores ($\beta = -2.29$, 95% CI: −4.35 to −0.24; $\beta = -3.03$, 95% CI: −6.05 to −0.01, respectively) at neonatal stage.	**
Parajuli et al., 2014<br><br>Nepal<br><br>Cross-sectional	N: 100<br><br>Age: At birth-6 months<br><br>Gender: 47 (47%) males<br><br>To investigate the effects of in utero exposure to heavy metals as well as the influence of the home environment on neurodevelopmental	Exposure: Highway air and water pollution<br><br>Sample: Umbilical cord blood<br><br>Method: Inductively coupled plasma mass spectrometer (ICP-MS)<br><br>Metal: Pb, As and Zn	Bayley Scale of Infant Development (BSID II)	There was no association between Pb levels and infant development scale scores at 6 months.	***

	scores in 6-month-old infants.				
Parajuli et al., 2015<br><br>Nepal<br><br>Longitudinal	N: 100<br><br>Age: 36.9 (0.4) months<br><br>Gender: 47 (47%) males<br><br>To examine the impact of in utero levels of toxic elements on neurodevelopment in children.	Exposure: Air pollution from highways, water and domestic environments<br><br>Sample: Umbilical cord blood<br><br>Method: Inductively coupled plasma mass spectrometer (ICP-MS)<br><br>Metal: Pb, As and Zn	Bayley Scale of Infant Development, Second Edition (BSID II)	Cord blood toxic element levels (Pb and As) were not associated with any developmental delay scores at 36 months.	***
Park et al., 2016<br><br>South Korea<br><br>Cross-sectional	ADHD sample: 114<br><br>Age: 8.79 (1.57) years<br><br>Gender: 83 (72.8%) males<br><br>Controls sample: 114<br><br>Age: 8.73 (1.65) years<br><br>Gender: 81 (71.1%) males<br><br>To investigate whether elevated blood Pb levels are a risk factor for ADHD.	Exposure: Not informed<br><br>Sample: Child blood<br><br>Method: Graphite furnace atomic absorption spectrometry<br><br>Metal: Pb	Kiddie-Schedule for Affective Disorders and Schizophrenia Present and Lifetime Version (K-SADS-PL-K)<br><br>Continuous Performance Test (CPT)	Children with ADHD showed significantly higher blood Pb concentrations compared to controls ($p = 0.003$). Total blood Pb concentration was associated with an increased risk of ADHD (odds ratio [OR] = 1.60, 95% CI: 1.04–2.45, $p < 0.05$).	**
Polanska et al., 2018<br><br>Poland<br><br>Longitudinal	N: 402<br><br>Age: At birth - 24 months<br><br>Gender: 195 (48.5%) males<br><br>To assess the association between prenatal exposure to low levels of Pb and neuropsychomotor development up to 24 months, considering sex differences in susceptibility.	Exposure: Not informed<br><br>Sample: Maternal and umbilical cord blood<br><br>Method: Triple Quadrupole Inductively Coupled Plasma Mass Spectrometer (ICP-QQQ)<br><br>Metal: Pb	Bayley Scales of Infant and Toddler Development-III (BSID III)	Higher umbilical cord blood Pb levels were associated with lower cognitive function scores in boys ($\beta = -2.07$, 95% CI = −4.07 to −0.06, $p = 0.04$).	**
Qiu et al., 2024<br><br>China<br><br>Longitudinal	N: 854<br><br>Age: 36 (0.3) months	Exposure: Not informed<br><br>Sample: Maternal urine	Bayley Scales of Infant and Toddler Development- III (BISD-III)	Higher levels of the metal mixture—vanadium (V), Cu, Zn, Sb, Ce, and U—were associated with a 2.37-fold increased risk of suboptimal gross motor development (95% CI: 1.15–4.86, $p = 0.012$).	***

	Gender: 416 (48.7%) males<br><br>To examine the associations between prenatal exposure to specific metals and metal mixtures and child neurodevelopment.	Method: Inductively Coupled Plasma Mass Spectrometry (ICP-MS)<br><br>Metal: Sb, As, Ba, Cd, Ce, Cs, Cr, Co, Cu, La, Pb, Mn, Hg, Mo, Ni, Re, Rb, Se, Sr, Tl, Sn, Ti, U, V, Zn.			
Rodrigues et al., 2016<br><br>Bangladesh<br><br>Longitudinal	N: 525<br><br>Age: 2.3 (1.7–3.3) years<br><br>Gender: 261 (47,9%) males<br><br>To investigate associations between environmental exposure to As, Mn and Pb and neurodevelopmental outcomes among children in Bangladesh.	Exposure: Water<br><br>Sample: Child blood<br><br>Method: LeadCare® II portable system II<br><br>Metal: Pb	Bayley Scales of Infant and Toddler Development, Third Edition (BSID-III)	Pb levels were negatively associated with cognitive development ($\beta = -0.17$).	***
Rosa et al., 2024<br><br>USA<br><br>Longitudinal	N: 326<br><br>Age: 6.53 (2.15) years<br><br>Gender: 182 (55.8%) males<br><br>To examine associations between prenatal metal mixtures and cognitive functioning in early and middle childhood.	Exposure: Not informed<br><br>Sample: Maternal urine<br><br>Method: Triple-quadrupole inductively coupled plasma–mass spectrometer (ICP-QQQ)<br><br>Metal: As, Cd, Mn, Pb and Sb	NIH Toolbox Cognition Battery (NIHTB-CB).	There was no evidence of an association between the mixture of metals and overall cognitive functioning scores in children.	**
Rothenberg et al., 2016<br><br>China<br><br>Longitudinal	N: 270<br><br>Age: At birth - 12 months<br><br>Gender: 127 (47%) males<br><br>To investigate associations between prenatal exposure to MeHg and offspring neurodevelopmental outcomes.	Exposure: Rice intake<br><br>Sample: Maternal blood and hair<br><br>Method: Atomic absorption spectrometry (AAS)<br><br>Metal: Hg and Pb	Bayley Scales of Infant Development (BSID)-II	Higher hair Hg concentration was negatively associated with the mental development index ($\beta = -4.9$). Higher blood Pb concentration was negatively associated with the psychomotor development index ($\beta = -11$).	***
Ruiz-Castell et al., 2012	N: 246<br><br>Age: 10.5-12.5 months	Exposure: Contaminated mining industries	Bayley Scales of Infant Development (BSID)	Maternal blood Pb levels were positively associated with mental development indices ($\beta = 2.27$, $p = 0.034$).	***

Bolivia<br><br>Longitudinal	Gender: 129 (52.44%) males<br><br>To investigate the association between prenatal exposure to trace metals and mental and psychomotor development in children at 11–12 months of age in a mining town in Bolivia.	Sample: Maternal blood<br><br>Method: Inductively Coupled Plasma Mass Spectrometry (ICP-MS)<br><br>Metal: Sb, As, Cd, Cs, Pb, Rb		Maternal blood cesium levels were positively associated with infant psychomotor development (β = 2.20, p = 0.015).	
Shah-Kulkarni et al., 2020<br><br>South Korea<br><br>Longitudinal	N: 523<br><br>Age: At birth - 6 months<br><br>Gender: 283 (54.1%) males<br><br>To explore the effects of prenatal exposure to a mixture of metals during pregnancy and at birth on neurodevelopment in 6-month-old infants.	Exposure: Not informed<br><br>Sample: Maternal and umbilical cord blood<br><br>Method: Atomic absorption spectrometer-graphite furnace<br><br>Metal: Cd and Pb	Korean version of Bayley Scales of Infant Development II (KBSID-II)	Exposure to mixtures of Pb, Hg, and Cd in early pregnancy and umbilical cord blood did not significantly affect mental development scores at 6 months. Exposure to mixtures of Pb, Hg, and Cd in early pregnancy and umbilical cord blood did not significantly affect psychomotor development scores at 6 months.	**
Shekhawat et al., 2021<br><br>India<br><br>Longitudinal	N: 167<br><br>Age: At birth - 6 months<br><br>Gender: 80 (48%) males<br><br>To assess the relationship between umbilical cord blood Pb concentration and early neurodevelopmental outcomes	Exposure: Not informed<br><br>Sample: Umbilical cord blood<br><br>Method: Inductively<br><br>Coupled Plasma Optical Emission Spectroscopy (ICP-OES)<br><br>Metal: Pb	The Bayley Scale of Infants Developments-III (BSID-III)	Umbilical cord blood Pb concentrations between 5.0 and 10.5 µg/dL were negatively associated with gross motor skills subscale scores (β = −0.29, 95% CI: −5.00 to 0.11, p = 0.042) at a mean age of 6.5 months.	***
Skogheim et al., 2021<br><br>Norway<br><br>Longitudinal	ADHD: 705<br><br>Age: From 2 years<br><br>Gender: 520 (73.8%) males<br><br>ASD: 397<br><br>Age: From 2 years<br><br>Gender: 336 (84.6%) males<br><br>Controls: 1034<br><br>Age: From 2 years	Exposure: Not informed<br><br>Sample: Maternal blood<br><br>Method: Inductively coupled plasma mass spectrometry (ICP-MS)<br><br>Metal: As, Cd, Cs, Pb and Hg.	Adult ADHD Self-Report Scale	The risk of ADHD in children exposed to the highest quartile of Cd compared to the lowest quartile was increased [OR = 1.59 (95% CI: 1.15, 2.18)]. Elevated risk of ASD was observed in children in the second quartile of As [OR = 1.77 (95% CI: 1.26, 2.49)] and in the highest quartiles of Cd [OR = 1.57 (95% CI: 1.07, 2.31)] and Mn [OR = 1.84 (95% CI: 1.30, 2.59)] compared to the first quartile (reference)	***

	Gender: 705 (68.2%) males<br><br>To investigate whether maternal levels of toxic metals and essential elements measured during mid-pregnancy, individually and in mixtures, are associated with childhood diagnoses of ADHD or ASD.				
Tatsuta et al., 2014<br><br>Japan<br><br>Longitudinal	N: 387<br><br>Age: 42.1 (40-45) months<br><br>Gender: 202 (52.2%) males<br><br>To investigate the impact of prenatal exposure to polychlorinated biphenyls (PCBs), MeHg, and Pb on the intellectual abilities of Japanese children at 42 months of age.	Exposure: Not informed<br><br>Sample: Umbilical cord blood<br><br>Method: Cold vapor atomic absorption spectrometry and inductively coupled plasma mass spectrometry (ICP-MS)<br><br>Metal: MeHg and Pb	Kaufman Assessment Battery for Children (K-ABC)	No significant associations were found between prenatal exposure to total Hg (THg) or Pb and children's intelligence scores.	***
Tatsuta et al., 2020<br><br>Japan<br><br>Longitudinal	N: 289<br><br>Age: 12 (11.1-12.8) years<br><br>Gender: 148 (51.21%) males<br><br>To examine the association between prenatal and postnatal Pb exposure and IQ at 12 years of age.	Exposure: Not informed<br><br>Sample: Umbilical cord and venous blood<br><br>Method: Inductively coupled plasma mass spectrometry (ICP-MS) and cold vapor atomic absorption spectrometry (CVAAS)<br><br>Metal: Hg and Pb	Wechsler Intelligence Scale for Children-Fourth Edition (WISC-IV)<br><br>Boston Naming Test (BNT)	Among boys, IQ was associated with child-blood Pb (Std β = −0,178) $p = 0.033$), but there was no association with Pb in cord blood ($B = -6.844$, $p = 0.309$). Umbilical cord blood Pb levels showed no significant correlation with any developmental outcomes among girls.	***
Taylor et al., 2018<br><br>United Kingdom<br><br>Longitudinal	N: 1558<br><br>Age: 7 years<br><br>Gender: 780 (50.06%) males<br><br>To investigate the association between prenatal exposure to Pb, Cd, and Hg, measured by maternal blood concentrations during	Exposure: Not informed<br><br>Sample: Maternal blood<br><br>Method: Inductively-coupled plasma mass spectrometry (ICP-MS)<br><br>Metal: Pb, Cd and Hg	ALSPAC Coordination Test	There was no evidence of associations between prenatal exposure to Pb, Cd, or Hg and motor skills measured at 7 years of age. Furthermore, no associations were found with probable developmental coordination disorder.	***

	pregnancy, and motor skills at 7 years of age.				
Tong et al., 2022<br><br>China<br><br>Longitudinal	N: 2164<br><br>Age: 55.6 (6.9) months<br><br>Gender: 1117 (51.6%) males<br><br>To explore the effect of prenatal exposure to serum Tl on cognitive development in preschool-aged children.	Exposure: Not informed<br><br>Sample: Maternal blood and umbilical cord blood.<br><br>Method: Inductively coupled plasma mass spectrometry (ICP-MS)<br><br>Metal: Tl	Wechsler Preschool and Primary Scale of Intelligence-Fourth Edition (WPPSI-IV)	Higher maternal serum Tl levels during pregnancy were associated with lower IQ in children (β = −1.51, 95% CI: −2.68 to −0.35, p = 0.01).	***
Tong et al., 2023<br><br>China<br><br>Longitudinal	N: 2164<br><br>Age: 4.6 (0.6) years<br><br>Gender: 1117 (51.6%) males<br><br>To investigate the impact of prenatal Ba concentrations on the intellectual function of preschool children using a birth cohort study.	Exposure: Not informed<br><br>Sample: Maternal blood and umbilical cord blood<br><br>Method: Inductively coupled plasma mass spectrometry (ICP-MS)<br><br>Metal: Ba, Pb and Tl	Chinese version of the Wechsler Preschool and Primary Scale of Intelligence-Fourth Edition (WPPSI-IV)	Elevated maternal serum Ba levels during pregnancy were significantly associated with reduced childhood intellectual function, as indicated by lower global IQ scores [−3.76 (95% CI: −6.19, −1.33)].	***
Valent et al., 2013<br><br>Italy<br><br>Longitudinal	N: 606<br><br>Age: 0-18 months<br><br>Gender: 307 (50.7) males<br><br>To analyze the association between prenatal Hg exposure through maternal fish consumption and polyunsaturated fatty acids with child neurodevelopment.	Exposure: Fish consumption<br><br>Sample: Maternal hair/blood and umbilical cord blood.<br><br>Method: Atomic absorption spectrometry, cold vapor atomic fluorescence detector (CVAFS) and Inductively coupled plasma–mass spectroscopy (ICP-MS).<br><br>Metal: Hg	Bayley Scales of Infant and Toddler Development - III (BSID-III)	Maternal total hair Hg levels were positively associated with language development scores in girls (β = 1.5291, p = 0.0445). Total Hg in maternal hair was not associated with positive scores on the language development scale in boys (β = 0.3551, p = 0.6278).	**
Valeri et al., 2017<br><br>India<br><br>Longitudinal	N: 825<br><br>Age: At birth - 40 months<br><br>Gender: 419 (50.78%) males	Exposure: Not informed<br><br>Sample: Umbilical cord blood	Bayley Scales of Infant and ToddlerDevelopment-III (BSID-III)	Higher levels of Mn and As were associated with decreased cognitive scores (β = −0.206, 95% CI: −0.39 to −0.02, p < 0.05).	***

	To evaluate the combined effect of intrauterine exposure to As, Mn, and Pb on child neurodevelopment.	Method: Coupled plasma mass spectrometer<br><br>Metal: As, Pb and Mn			
Vejrup et al., 2017<br><br>Norway<br><br>Longitudinal	N: 2239<br><br>Age: At birth - 5 years<br><br>Gender: 1187 (53%) males<br><br>To assess the associations between total Hg concentration in maternal blood during pregnancy and dietary exposure to MeHg with language and communication development	Exposure: Seafood consumption<br><br>Sample: Maternal blood<br><br>Method: Cold vapor atomic fluorescence spectrophotometry and inductive couple plasma mass spectrometry (ICP-MS)<br><br>Metal: Hg and Se	Ages and Stages Communication scale (ASQ)<br><br>Speech and Language Assessment Scale (SLAS)<br><br>Language-Related Difficulties list (language 20)	The analysis showed that maternal blood Hg concentration was not significantly associated with language and communication scale scores.	***
Xue et al., 2020<br><br>China<br><br>Longitudinal de caso controle	N: 456<br><br>Dyslexic: 228<br><br>Age: 9.76 (1.29) years<br><br>Gender: 171 (75%) males<br><br>Non-dyslexic: 228<br><br>Age: 9.69 (1.25) years<br><br>Gender: 171 (75%) males<br><br>To investigate the association between urinary metal concentrations and dyslexia among children	Exposure: Not informed<br><br>Sample: Child urine<br><br>Method: Inductively coupled plasma mass spectrometry (ICP-MS)<br><br>Metal: Ag, As, Al, Ba, Cd, Cs, Cr. Co, Cu, Hg, I, Pb, Mn, Ni, Rb, Se, Sr, Ti, U, V and Zn	Dyslexia Checklist for Chinese Children (DCCC)<br><br>Pupil Rating Scale-Revised Screening (PRS)	Children with dyslexia showed higher concentrations of Sr ($p = 0.028$), Ag ($p = 0.014$), and U ($p = 0.005$).	***

Good quality: ***, Fair quality: ** and Poor quality: *
